# Associations between neighbourhood walkability and daily steps in adults: a systematic review and meta-analysis

**DOI:** 10.1186/s12889-015-2082-x

**Published:** 2015-08-11

**Authors:** Samantha Hajna, Nancy A. Ross, Anne-Sophie Brazeau, Patrick Bélisle, Lawrence Joseph, Kaberi Dasgupta

**Affiliations:** Department of Epidemiology, Biostatistics and Occupational Health, McGill University, 1020 Pine Avenue West, Montréal, QC Canada; Department of Geography, McGill University, 805 Sherbrooke Street West, Montréal, QC Canada; Department of Medicine, Division of Clinical Epidemiology, McGill University Health Centre, 687 Pine Avenue West, Montréal, QC Canada

**Keywords:** Neighbourhood walkability, Daily step count, Walking, Environments, Physical activity

## Abstract

**Background:**

Higher street connectivity, land use mix and residential density (collectively referred to as neighbourhood walkability) have been linked to higher levels of walking. The objective of our study was to summarize the current body of knowledge on the association between neighbourhood walkability and biosensor-assessed daily steps in adults.

**Methods:**

We conducted a systematic search of PubMed, SCOPUS, and Embase (Ovid) for articles published prior to May 2014 on the association between walkability (based on Geographic Information Systems-derived street connectivity, land use mix, and/or residential density) and daily steps (pedometer or accelerometer-assessed) in adults. The mean differences in daily steps between adults living in high versus low walkable neighbourhoods were pooled across studies using a Bayesian hierarchical model.

**Results:**

The search strategy yielded 8,744 unique abstracts. Thirty of these underwent full article review of which six met the inclusion criteria. Four of these studies were conducted in Europe and two were conducted in Asia. A meta-analysis of four of these six studies indicates that participants living in high compared to low walkable neighbourhoods accumulate 766 more steps per day (95 % credible interval 250, 1271). This accounts for approximately 8 % of recommended daily steps.

**Conclusions:**

The results of European and Asian studies support the hypothesis that higher neighbourhood walkability is associated with higher levels of biosensor-assessed walking in adults. More studies on this association are needed in North America.

## Background

Global rates of overweight and obesity are on the rise [1]. Although there have been small successes in the treatment and prevention of these conditions, no country has yet managed to reverse its epidemic [2]. This was highlighted in a series of six papers that were released in the February 2015 edition of the *Lancet* [2]. To more effectively combat the rising rates of obesity and obesity-related complications, interventions that acknowledge the interacting roles of individuals and their environments are needed [1–3]. Since the late 1990′s there has been growing interest in the role of neighbourhood environments on obesogenic behaviour [4–7]. The hypothesis is that the adoption of positive health behaviours will only be possible given choice-enabling environments [4, 8]. For example, in neighbourhoods with higher densities of fast food outlets, residents are more likely to consume fast food products than residents living in neighbourhoods where these outlets are not as prominent [9, 10]. One area of growing interest is on the role of neighbourhood designs on physical activity behaviour.

Street connectivity, land use mix and residential density are three large-scale features of neighbourhood designs that are commonly studied for their associations with physical activity [11–14]. ***Street connectivity*** is defined as the number of three or more-way intersections per square kilometre within a neighbourhood buffer, where a greater number of intersections is indicative of increased ease of movement between origins (e.g., residences) and destinations (e.g., shops and parks) [12, 15]. Neighbourhoods with higher intersection densities are typically designed using finer grid patterns and thus provide more straight-line options for travelling between origins and destinations [15]. In addition to this, such neighbourhoods slow traffic as a result of multiple stopping sites and allow pedestrians to reach their destinations via a variety of routes, making non-motorized transport more appealing [13]. ***Land use mix*** is a measure of the number of different types of land uses in a neighbourhood [12, 15]. Many downtown neighbourhoods have a large land use mix. Apartments are located above street-level shops and in close proximity to churches, schools and other services making it convenient for residents to walk to these locations [13]. This is in contrast to many newer suburban neighbourhoods where wide separation between residential and commercial land makes motorized transportation to points of interest a near necessity [15]. There are several ways to calculate land use mix [16]. The most common method is using the Shannon entropy score [13, 17]. The score ranges from 0 to 1 where a higher score is indicative of greater heterogeneity in land uses within a neighbourhood [12]. ***Residential density*** is defined as the number of residences per square kilometer of residential land area in the home buffer [14] or per square kilometer of the household’s dissemination block [12]. Neighbourhoods with greater residential densities are generally more conducive to non-motorized transport as a result of there being more people to visit and a greater demand for accessible community services, such as shops and parks [15]. Street connectivity, land use mix and residential density are correlated [18]. As a result, when estimating their associations with health outcomes, researchers commonly aggregate these measures into an index that captures neighbourhood walkability – that is, the degree to which a neighbourhood is “walking friendly” [13, 18].

Higher neighbourhood walkability has been linked to higher levels of utilitarian walking (i.e., walking for specific purposes such as for travelling to school or to the grocery store) [19–22]. The findings are weaker or non-existent for leisure-time walking, suggesting that neighbourhood designs may not be important drivers of this type of physical activity [21, 22]. While utilitarian and leisure-time walking - two components of overall physical activity - are well studied, our understanding of the association of walkability with total walking is limited. Since total walking is arguably the more salient correlate of improved health outcomes [23–26], understanding its association with neighbourhood walkability is of particular interest.

Distinguishing between subtypes of physical activity (e.g., utilitarian and leisure-time walking), necessitates reliance on self-report. In contrast, total walking may be assessed using biosensors (i.e., pedometers or accelerometers). Daily steps as captured by biosensors provide a good estimate of total walking [27, 28]. Few studies, however, have examined the association between walkability and biosensor-assessed total walking [5]. The objective of the present study was to summarize the current body of knowledge on the association between neighbourhood walkability (based on street connectivity, land use mix, and/or residential density) and total walking (as captured by the daily step count function of biosensors) in adults.

## Methods

### Search strategy

The systematic review was conducted in compliance with the Preferred Reporting Items for Systematic Reviews and Meta-Analyses (PRISMA) guidelines [29]. A systematic search was conducted on titles, abstracts, keywords, MeSH terms and/or subject headings, as appropriate, that were ever indexed in PubMed, SCOPUS, or Embase (Ovid) prior to May 20, 2014 (i.e., from 1946 for PubMed, from 1996 for SCOPUS, and from 1996 for Embase (Ovid)). The following search string was used: [physical activity OR walk OR walking OR pedometer OR acceleromet* OR exercise OR actigraphy OR actimetry] *AND* [built environment OR walkable OR walkability OR street connectivity OR land use mix OR residential density OR population density OR environment planning OR neighborhood OR home environment OR urban design OR environment design OR residence characteristics OR Geographic Information Sys* OR geographic mapping]. The search strategy was developed by SH in consultation with a librarian from the Royal Victoria Hospital (Montreal, Quebec, Canada).

### Article review and data extraction

All of the identified articles were compiled in Endnote (×4.0.2). Two independent reviewers (SH and AB) reviewed all of the titles and abstracts. The following inclusion criteria were applied: 1) study population ≥18 years, 2) the objective of the study was to estimate the associations between street connectivity, land use mix, and/or residential density (derived using Geographic Information Systems (GIS)) and pedometer or accelerometer-assessed daily steps, 3) effect estimates were reported, and 4) the article was published in English. Data were abstracted using a standardized form (SH). Abstracted information included study population, sample size, exposure and outcome measurement, and a summary of the reported effect estimates.

### Statistical analysis

Confidence intervals (95 %) were not presented in the original papers and were calculated for the purposes of this analysis based on the reported information. Only the studies that reported differences in mean steps taken per day between high and low walkability neighbourhoods were included in the meta-analysis. The differences in means were pooled using a Bayesian normal-normal hierarchical model. At the first level of this model we assumed that the means within each group from each study followed normal densities, with study specific means [i, j], i = 1, 2, 3, 4 indexing the studies and j = 1,2 indexing the groups. We similarly assumed that the logarithms of standard deviations within each group from each study followed normal densities. At the second level of the hierarchical model, the study specific means within each of the two groups were again assumed to follow a normal density, with a global mean representing the overall mean within each group across studies, and a global variance parameter representing the spread of these means across the studies within each group. Similarly, the log standard deviations followed normal densities with the global mean representing the overall means of the log standard deviations, and the variance parameter indicating the spread of these values across studies within each group. We used normal (8000, 100,000) and uniform (0, 600) prior densities for the global means and log (SD), respectively. WinBUGS was used to run the hierarchical model (Version 1.4.3, MRC Biostatistics Unit, Cambridge UK). The forest plot was produced in R (CRAN, Version 3.0.1).

## Results

### Search results

The search that was conducted on articles published prior to May 20, 2014 yielded a total of 8,744 unique abstracts. After title and abstract review, a total of 30 articles were identified and underwent full article review. Of these, six met the inclusion criteria.

### Qualitative analysis

Four of the six studies were conducted in Europe (Belgium [30, 31], Czech Republic [32], and Scotland [33]) and two of the six studies were conducted in Asia (China [34] and Japan [35]) (Table 1). All of the studies were cross-sectional. The measurement of daily steps and neighbourhood walkability was comparable across all studies. Daily steps were assessed using biosensors that have been validated for use in adults [36–42]. Neighbourhood walkability was assessed using comparable operational definitions of street connectivity, land use mix, and/or residential density. In three studies the authors predefined high and low walkable neighbourhoods and randomly selected participants from these areas [30, 31, 35]. In the remaining three studies [32–34], neighbourhood walkability was defined for each participant after they were selected into the study.

**Table 1:** Previous studies on the associations between GIS-derived walkability and daily steps in adults

						Overall Walking				Utilitarian Walking			
1st Author, Publication Date	N	Age	Location	Sampling Design	Neighbourhood Walkability Measurement (*cut*-*off for high vs. low walkability)*	Measurement	Findings	Association^a^	Difference in mean steps per day for people living in high versus low walkability neighbourhoods (95 % confidence interval) ^b^	Measurement	Findings	Association^a^	Difference in mean time walking for utilitarian purposes for people living in high versus low walkability neighbourhoods (95 % confidence interval) ^b^
Kondo, 2009	112	30 to 69	Hagi City, Japan	Sampling from high and low walkable neighbourhoods using a stratified random sampling method based on sex and 5-year age strata.	GIS-derived walkability based on street connectivity, residential density, land use mix *(not specified)*	Accelerometer	High walkability: 9364 steps/day; SE 567	INC	1071 steps/day (95 % CI −399 to 2540)	Min/day (IPAQ)^c^	High walkability: 3.3 min/day; SE = 2.1	0	−5 min/day (95 % CI −10 to 1)
							Low walkability: 8294 steps per day; SE 491				Low walkability: 8.0 min/day; SE = 2.0		
Van Dyck, 2011	350	42.4 ± 13.2	Flanders, Belgium	Sampling from high and low walkable neighbourhoods based on address list provided by the local government.	Urban *vs.* rural neighbourhoods based on GIS-derived walkability based on street connectivity and population density *(not specified)*	Pedometer	High walkability: 9323 steps/day; SD 3473	INC	548 steps/day (95 % CI −230 to 1326)	Min/week (NPAQ)^c^	High walkability: 97.5 min/week; SD = 96.4	**+**	76 min/week (95 % CI 58 to 94)
							Low walkability: 8775 steps per day; SD 3942				Low walkability: 21.9 min/week; SD = 72.3		
Dygryn, 2010	70	20 to 64	Olomouc, Czech Republic	Random selection of participants in city. Walkability was determined after inclusion into the study.	GIS-derived walkability based on street connectivity, residential density, floor area ratio, land use mix *(upper* versus *lower 5 deciles)*	Pedometer	High walkability: 11318 steps/day; SD 4091	**+**	2088 steps/day (95 % CI 440 to 3736)	n/a	n/a	n/a	n/a
							Low walkability: 9230 steps per day; SD 2554						
Van Dyck, 2009	120	20 to 65	Sint-Niklaas, Flanders (Belgium)	Sampling from high and low walkable neighbourhoods. Letters of invitation sent to randomly selected people. Letters were followed up with house visits to recruit people.	Two neighbourhoods with greatest contrast in GIS-derived walkability based on street connectivity and residential density (*not specified*)	Pedometer	High walkability: 9318 steps/day, SD 3055	+	1222 step/day (95 % CI 131 to 2313)	Min/week (NPAQ)^c^	High walkability: 104.33 min/week; SD = 95.1	**+**	82 min/week (95 % CI 53 to 110)
							Low walkability: 8096 steps per day; SD 3044				Low walkability: 22.83 min/week; SD = 61.0		
Robertson, 2012	76	27 to 66	Glasgow, Scotland	Sampling of people from Glasgow who were low active and part of low socioeconomic groups. Advertisement for participation was made in public locations (e.g., shops). Walkability was determined after inclusion into the study.	GIS-derived commercial and residential land use mix	Pedometer	A one-unit increase in land use mix (from no mix to a perfect mix) was associated with:			n/a	n/a	n/a	n/a
							1896 more steps/day (SE = 583) at *6-months* post community intervention	+	1896 steps/day(95 % CI 754 to 3038)				
							1260 more steps/day (SE = 622) at *12-months* post community intervention	+	1260 steps /day(95 % CI 40 to 2479)				
Zhang, 2014	1,100	46 to 80	Shanghai, China	Stratified random samples based on even distribution of community types. Selected households were sent letters of invitation. Walkability was determined after inclusion into the study.	GIS-derived street connectivity	Pedometer	Living in a neighbourhood one-SD above the mean street connectivity was associated with accumulating 21 more steps/day *(no variance estimates reported)*	Unknown based on reported information	Confidence intervals around the linear regression estimate could not be calculated based on the information reported in the text.	n/a	n/a	n/a	n/a

^a^Positive relationship (+); negative relationship (−); INC (inconclusive; more research is needed to better estimate this effect); 0 (no effect)

^b^95 % confidence intervals were recalculated based on information reported in the original manuscripts (i.e., group sample sizes, standard deviations/standard errors, and/or p-values)

^c^International Physical Activity Questionnaire (IPAQ); Dutch Version of the Neighbourhood Physical Activity Questionnaire (NPAQ)

The point estimates of all six studies suggested that higher walkability was associated with a greater number of daily steps. Based on the confidence intervals, these associations were conclusive for only three of these studies [30, 32, 33]. In addition to examining the role of walkability with daily steps, three of the six studies assessed the role of walkability on utilitarian walking [30, 31, 35]. In the two studies from Belgium, adults living in high walkable neighbourhoods spent over 75 minutes more per week in utilitarian walking (76 minutes, 95 % CI 58 to 94 [31]; 82 minutes, 95 % CI 53 to 110 [30]) compared to people living in low walkable neighbourhoods. In the Japanese study, adults living in high walkable neighbourhoods reported walking 5 min per day less for utilitarian purposes than people living in low walkable neighbourhoods (95 % CI −10 to 1) [35].

### Meta-analysis

**Fig. 1:**
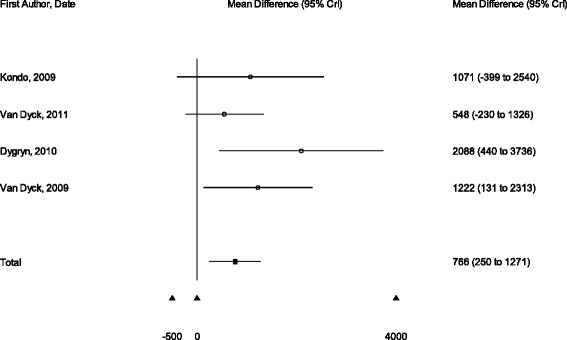
*Short Title*: Forest plot of the results of previous studies on walkability and daily steps. *Detailed Legend*: Forest plot of the results of the previous studies that have been conducted on the association between Geographic Information Systems-derived measures of walkability (i.e., street connectivity, land use mix, and/or residential density) and pedometer and/or accelerometer-assessed steps per day in adults. The estimates represent the mean differences in daily steps between high and low walkability neighbourhoods (95 % credible intervals)

The results of four studies could be pooled in a meta-analysis given that comparable effect measures were reported (i.e., the mean differences in steps/day between high and low walkability neighbourhoods) [30–32, 35]. The confidence intervals in the Belgian and Czech studies demonstrated clear positive associations of walkability with steps (Belgium [30]: 1222 steps per day, 95 % CI 131 to 2313; Czech Republic [32]: 2088 steps per day, 95 % CI 440 to 3736). Those in the Japanese study and the second Belgian study precluded definitive conclusions (Japan [35]: 1071 steps per day, 95 % CI −399 to 2540; Belgium [31]: 548 steps per day, 95 % CI −230 to 1326). A meta-analysis of these results demonstrated that participants living in high compared to low walkable neighbourhoods accumulated 766 more steps per day (95 % credible interval (CrI) 250 to 1271) (Fig. 1).

## Discussion

Six studies have examined the association of GIS-derived street connectivity, land use mix, and/or residential density with pedometer or accelerometer-assessed daily steps in adults. Based on a meta-analysis of the results reported in four of these studies, living in high compared to low walkable neighbourhoods is associated with accumulating 766 more steps per day. This is on par with the seasonal deficits in daily steps that have been documented in the literature [25, 43, 44].

The majority of evidence in support of neighbourhood walkability as a correlate of higher levels of physical activity comes from studies that rely on self-reported measures of physical activity [5, 45]. Recent reviews have summarized these findings, but other than suggesting that walkability is likely associated with higher levels of physical activity, these reviews do not quantify the association [5, 46–49]. Our study is the first to quantify the association between walkability and biosensor-assessed total walking in adults. All of the studies that were retained in the meta-analysis assessed steps using tools that have been validated for use in adults – thereby increasing the comparability of the outcomes across the studies. Daily step count as a measure of physical activity has several valuable properties. First, it is an accurate and easily understood measure of physical activity [27, 28]. Daily steps are more easily interpreted than accelerometer-assessed moderate-to-vigorous intensity physical activity [26]. Furthermore, there are established cut points for activity levels based on daily steps (i.e., sedentary: <5,000 steps/day, low active: 5,000 to 7,499 steps/day, somewhat active: 7,500 to 9,999 steps/day, active: 10,000 to 12,499 steps/day, highly active: ≥12,500 steps/day) [27] and pedometer-based step count interventions have been effective in facilitating increases in walking among adults [50, 51].

Some study limitations should also be noted. First, the pooled estimates are based on a relatively small number of studies. To build our understanding of the role of neighbourhood designs on walking in adults, more studies using comparable exposure and outcome measurements are needed. Second, although the operational definitions of street connectivity, land use mix and residential density were highly comparable across studies, some variability in measurement is expected due to between-country differences in actual walkability [14] and in the quality of the spatial data that were used to calculate walkability. However, the bias arising from this variability is offset given that we pooled relative (i.e., high compared to low walkable neighbourhoods) rather than absolute estimates of walkability (i.e., actual residential density). Third, we only estimated the associations between walkability and daily steps as defined by three large-scale features of neighbourhood designs (street connectivity, land use mix, residential density). Daily steps may be associated with other components of walkability, such as neighbourhood safety, presence of amenities, and social cohesion. Research on the associations of these features with daily steps is encouraged as a means of building our understanding of the role that environments have on the total levels of physical activity that adults achieve.

## Conclusions

Our analysis suggests that living in high compared to low walkable neighbourhoods is associated with accumulating 766 more steps per day. Given that accumulating at least 10,000 steps per day is recommended for healthy adult populations [26], this is equivalent to approximately 8 % of recommended daily steps. While there is consistent evidence that higher neighbourhood walkability is associated with higher levels of biosensor-assessed walking in Europe and possibly in Asia, no comparable studies have been conducted in North America. Given higher levels of car ownership [52] and physical inactivity [53] in North America, the association between neighbourhood designs and the total amount of walking that people achieve may be different in this context. To increase our understanding of this relationship in North America, more studies using comparable measures of exposures and outcomes in this setting are needed.

## Abbreviations

CI: Confidence interval
CrI: Credible interval
